# Influence of Gender Bias on Distribution of Hybrid Sterility in Rice

**DOI:** 10.3389/fpls.2022.898206

**Published:** 2022-07-12

**Authors:** Zin Mar Myint, Yohei Koide

**Affiliations:** Laboratory of Plant Breeding, Research Faculty of Agriculture, Hokkaido University, Sapporo, Japan

**Keywords:** rice, hybrid sterility, selfish genes, reproductive isolating barrier, transmission ratio distortion

## Abstract

Hybrid sterility genes define species identities, setting reproductive barriers between distantly related *Oryza* relatives. They induce allelic-specific selective gametic abnormalities by killing pollens, embryo sacs, or both, and thus resulting in the male specific transmission ratio distortion (*m*TRD), female specific transmission ratio distortion (*f* TRD), and/or sex-independent transmission ratio distortion (*si*TRD) in hybrids. Although more than 50 hybrid sterility genes have been reported, comprehensive analysis on the distributional pattern of TRD systems in *Oryza* species is limited. In this review, we surveyed the TRD systems and the underlying possible mechanisms in these species. In rice, pollen killers which cause *m*TRD are often observed in higher frequency than egg killers and gamete eliminators, which are factors affecting *f* TRD and *si*TRD, respectively. Due to the rather massive population of pollen grains, their reduction in the number caused by hybrid sterility possesses a smaller selective disadvantage to the hybrid individuals, in contrast to female gamete abortion. The pattern of TRD distribution displays less abundancy in *si*TRD. It suggests that fixation of *si*TRD might require a certain time rather than single sex-specific factors. The presence of linked sterility factors worked for *m*TRD and *f* TRD, and strength of their linkage in chromosomal regions might determine the type of sterility and TRD. The study of TRD systems has a potential to reveal the relationships between selfish genes and their functions for reproductive isolation.

## Introduction

Based on interbreeding, the biological species concept stated, “Species are groups of interbreeding natural populations that are reproductively isolated from other such groups.” (Mayr, 1996; Coyne and Orr, 2004). It literally means that the boundaries between the population of one species and that of another are defined by reproductive barriers, and the mechanism itself is referred to as “reproductive isolation”, in which genetically based intrinsic barriers prevent gene flow between populations of different species (Nosil, 2013). Reproductive isolation maintains species identity, and evolutionary biologists are highly interested in this area of study. Reproductive isolation is considered to be incidentally acquired as a by-product of other divergences between species' populations (Nosil, 2013). The genetic variations gradually increase over time, and the accumulation of those changes and divergences is probably either neutral or wholesome to its own genetic background while it works as a deleterious factor in other alternative genetic backgrounds, exclusively in a heterozygous state (Turelli, 1998). It is possible for hybrid dysfunction to appear due to a divergence in multiple genomic regions across the whole genome in a wide variety of species populations. The Bateson-Dobzhansky-Muller (BDM) model is a model in which negatively epistatic interaction between divergent alleles contributes deleterious effects within hybrid populations (Bateson, 1909; Dobzhansky, 1937; Muller, 1942; Kubo et al., 2008; Nosil, 2013; Xie et al., 2019a) and is a widely accepted model of the emergence of reproductive barriers. However, are genes for reproductive isolation always neutral or wholesome when they spread in a population? By summarizing genes for hybrid sterility, a form of reproductive barrier, we will discuss how selfish genes act to build species barriers in rice.

## Reproductive Barriers in Rice

In plants, reproductive barriers can be basically divided into two categories, namely prezygotic reproductive isolation and postzygotic reproductive isolation, based on the developmental stage in which they give rise (Koide et al., 2008b; Ouyang and Zhang, 2013; Zin Mar et al., 2021). While prezygotic isolation occurs during the formation of the zygote, the latter restricts the introgressive gene flow in crossed populations, inducing hybrid arrest after fertilization at different developmental stages and/or other advanced generations (Chen et al., 2016). Hybrid sterility, the gametic disorder at their reproductive stage with the failure to produce fertile male and/or female gametes in normally grown hybrid plants, is one form of postzygotic reproductive isolation. The genus *Oryza*, which consists of two cultivated rice species and 22 wild rice relatives (Ammiraju et al., 2010), is a valuable pool for improvement of agricultural traits. In rice genetics, interspecific and intraspecific hybrid sterility, which is one of the most pronounced forms of postzygotic reproductive isolation, has been the most extensively investigated subject across a wide variety of genomic regions in a substantial number of different rice populations (Ouyang and Zhang, 2013; Li et al., 2020).

## The Genetic Models of Hybrid Sterility

Based upon either sporophytic or gametophytic function and the number of loci involved, altogether four separate genic models underly rice hybrid sterility: namely (1) One-locus sporo-gametophytic interaction, (2) Duplicate gametic lethals, (3) One-locus sporophytic interaction, and (4) Complementary sporophytic interaction (Oka, 1957; Sano et al., 1979; Koide et al., 2008b). Among them, one-locus sporo-gametophytic interaction fits with genetic mechanisms for the majority of sterility loci in rice (Oka, 1957; Xie et al., 2019a). Therefore, we will focus on single or tightly linked sterility loci in this review (see Table 1). Hybrid sterility loci, such as *S1, S2*, and *S5*, can perfectly explain how one-locus sporo-gametophytic model works in rice sterility (Sano et al., 1979; Chen et al., 2009; Yang et al., 2012, 2016; Xie et al., 2017; Koide et al., 2018; Zin Mar et al., 2021). As an example, for *S2* locus, although *S2*^*g*^ (allele derived from *oryza glaberrima*) and *S2*^*s*^ (allele derived from *O. sativa*) are neutral in their respective backgrounds, the incompatible interaction between these two alleles occurred in heterozygous hybrids. As a result, neither male nor female gametes which carry *S2*^*g*^ allele survive, causing preferential transmission of selfish allele *S2*^*s*^ in later generations (Sano et al., 1979; Zin Mar et al., 2021). Such a preferential transmission of one of the two alleles is referred to as transmission ratio distortion (TRD). Selfish genes causing TRD take advantage over their alternative alleles, and preferentially promote their own distribution in populations, with the help of incompatibilities that occur in heterozygous hybrids.

**Table 1:** Hybrid sterility loci in rice and their sex-specificity-related transmission ratio distortion.

**Sex-specific transmission ratio distortion (TRD)**		**Loci**	**References**
*m*TRD	Interspecific cross	*S3*	Sano, 1983
		*S12*(*t*)	Sano, 1994
		*S13*	Koide et al., 2007
		*S18*	Doi et al., 1998
		*S19*	Taguchi et al., 1999
		*S20*	Doi et al., 1999
		*S21*	Miyazaki et al., 2007
		*S22A* and *S22B*	Sakata et al., 2021
		*S23*(*t*)	Fang et al., 2019
		*S27*	Yamagata et al., 2010
		*S28*	Yamagata et al., 2010
		*S29*(*t*)	Hu et al., 2006
		*S34*(*t*)	Zhang et al., 2005
		*S36*	Win et al., 2009
		*S38*	Xu et al., 2014
		*S39*	Xu et al., 2014
		*S44*	Zhao et al., 2012
		*S51*	Li et al., 2018
		*S52*	Li et al., 2018
		*S53*	Li et al., 2018
		*S54*	Li et al., 2018
		*S55/qHMS7*	Li et al., 2018
			Yu et al., 2018
		*S56*	Zhang et al., 2018
	Intrapecific cross	*S14(t)*	Sano, 1994
		*S24*(*t*)	Kubo et al., 2000
		*S25*(*t*)	Kubo et al., 2001
		*S35*	Kubo et al., 2016
		*Sa*	Long et al., 2008
		*Sb*	Li et al., 2006
		*Sc*	Shen et al., 2017
		*Sd*	Guiquan et al., 1994
		*Se*	Guiquan et al., 1994
		*Sf*	Guiquan et al., 1994
*f*TRD	Intraspecific cross	*S5*	Yang et al., 2012
		*S-7*	Yu et al., 2016
		*S-8*	Wan et al., 1993
		*S-9*	Wan et al., 1996
		*S15*	Wan et al., 1996
		*S16*	Wan and Ikehashi, 1995
		*S32*(*t*)	Li et al., 2005
		*S29(t)*	Zhu et al., 2005b
		*S30(t)*	Zhu et al., 2005a
*si*TRD	Interspecific cross	*S1*	Xie et al., 2019b
		*S2*	Zin Mar et al., 2021
		*S6*	Koide et al., 2008a
		*S33*(*t*)	Ren et al., 2005
		*S37*	Xu et al., 2014
	Intraspecific cross	*S10*	Sano et al., 1994
		*S11*(*t*)	Sawamura and Sano, 1996

## Sex-Specificity of Trd in Hybrid Sterility in Rice

**Figure 1:**
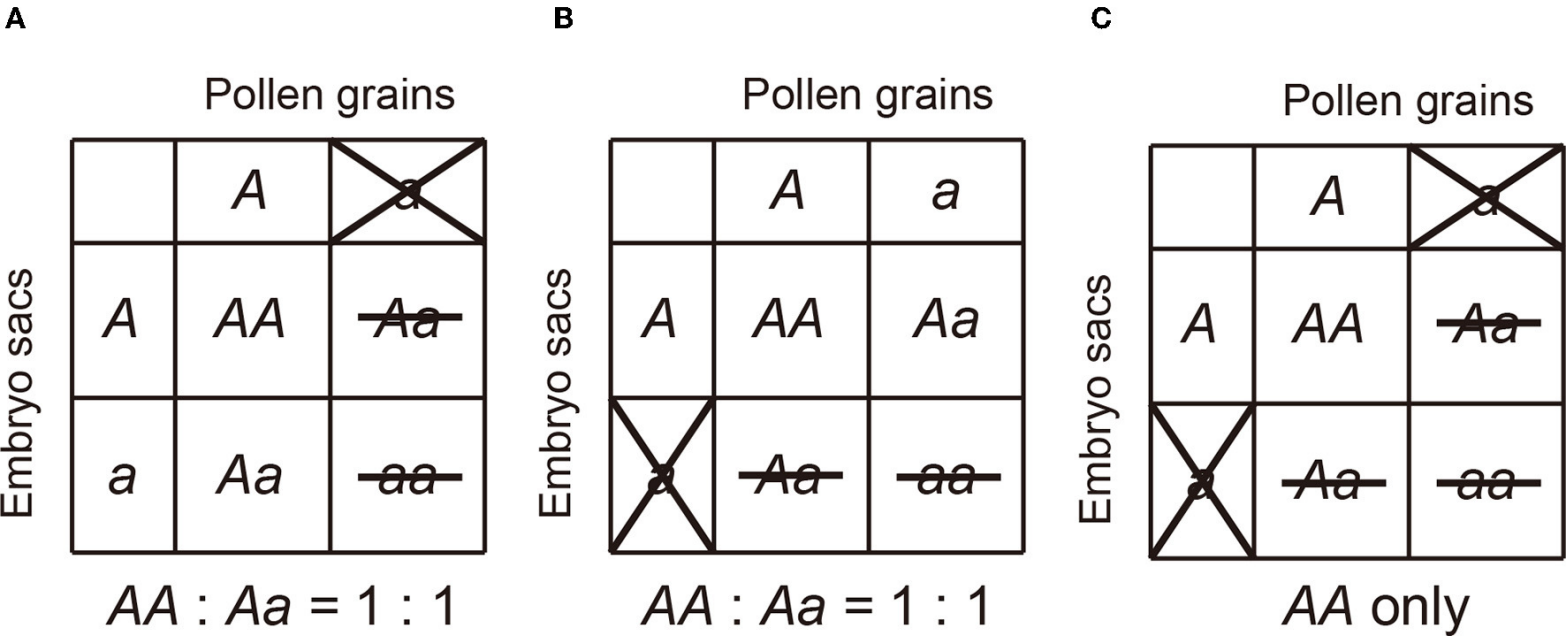
The genetic actions of three categories of hybrid sterility in rice. **(A)** Pollen killer, **(B)** Egg killer, **(C)** Gamete eliminator.

Although several underlying mechanisms behind the TRD phenomenon, including non-random segregation of chromosomes during meiosis (Pardo-Manuel de Villena and Sapienza, 2001; Birchler et al., 2003; Fishman and Willis, 2005; Koide et al., 2008a), unequal gametic success in fertilization (Price, 1997; Diaz and Macnair, 1999; Seymour et al., 2019), and embryo lethality (Lyttle, 1991; Silver, 1993; Price, 1997; Diaz and Macnair, 1999; Ubeda and Haig, 2005; Moyle, 2006) has been reported in plants, selective gametic abnormality (Lyttle, 1991; Silver, 1993; Ubeda and Haig, 2005; Moyle, 2006; Koide et al., 2008a) caused by hybrid sterility locus is the most frequently observed in *Oryza* species. The selective abnormality occurs in either male/female-gametes or sex-independently. In this report, the former and latter are termed *m*TRD (male specific transmission ratio distortion)/*f* TRD (female specific transmission ratio distortion) and *si*TRD (sex-independent transmission ratio distortion), respectively, (Maguire, 1963; Sano, 1983; Koide et al., 2008c; Ouyang and Zhang, 2013). Therefore, in general, hybrid sterility genes in rice can be divided into three subcategories: pollen killer (PK: which induces hybrid male sterility and *m*TRD), egg killer (EK: which results in hybrid female sterility and *f* TRD), and gamete eliminator (GE: which eliminates both pollen and embryo sac causing *si*TRD) (Figure 1).

Several loci causing TRD have been cloned and their underlying mechanisms have been studied. The *S5* locus is the most renowned hybrid sterility locus in the study of rice reproductive barriers. This locus is an example of “Killer-Protector system”, and its effect is shown in the failure of embryo sac fertility (We note that “Killer” in the Killer-Protector system and “Killer” in PK or EK have different actions. The former “Killer” induces the abortion of gametes with both alleles. The “Protector” selectively protects gametes with one of two alleles from the “Killer” function. As a result, the preferential dysfunction of gametes with one of two alleles occurs. The factor causing preferential function is referred to as PK, EK, or GE. Thus, PK and EK is a concept of a factor with the combined function of “Killer” and “Protector”.). Three different tightly linked genes (*ORF3, ORF4*, and *ORF5*) regulate the tripartite complex in indica-japonica crossed species. The indica-derived *S5* allele (*ORF3*+, *ORF4*–, and *ORF5*+) contained both functionally active Protector and Killer genes, *ORF3*+ and *ORF5*+, while its allelic japonica-derived *S5* region (*ORF3*–, *ORF4*+, and *ORF5*–) carries inactive Protector and Killer genes, *ORF3*– and *ORF5*–. However, the indica-derived Killer factor *ORF5*+ enacts its killing function with the assistance of its japonica-derived partner *ORF4*+ in the heterozygous combination. Meanwhile, *ORF3*+, which derives from the indica, selectively protects the embryo sac in which it resides. In the absence of stress responsive gene *ORF3*+, the unsolved endoplasmic reticulum (ER) stress induced by *ORF5*+ in partner with *ORF4*+, triggers programmed cell death (PCD), which results in embryo sac abortion (Yang et al., 2012).

The *S1* locus is one of the major reproductive barriers between Asian and African rice species and a remarkable *si*TRD locus. The *O. glaberrima* allele (*S1*^*g*^) takes advantage over *O. sativa* allele (*S1*^*s*^) within populations, thus preferentially transmitting *S1*^*g*^ and eliminating both male and female gametes when two alleles meet in a heterozygous state (*S1*^*s*^*/S1*^*g*^) (Koide et al., 2008c, 2018). According to Xie et al. (2017), CRISPR/Cas9-generated knockout mutants of *OgTPR1* on *S1*^*g*^ region (which encodes a protein with two trypsin-like peptidase domains and one ribosome biogenesis regulatory protein domain) produce normally fertile pollen and embryo sacs in crossing to *O. sativa* parent. The tripartite gamete Killer-Protector complex involving *S1A4, S1TPR* (*OgTPR1*), and *S1A6* (*SSP*) of the *S1*^*g*^ region, generates a sterility signal in sporophytic cells and protect itself with *S1TPR* in gametophytic cells, whereas its allelic *S1*^*s*^ region lacks both functional killer and protector (Xie et al., 2019b).

The evolution of Killer-Protector system should be explained by models in which the deleterious effect of the Killer did not occur in a lineage (e.g., BDM model or parallel-sequential divergence model, Ouyang and Zhang, 2013), because without Protector, Killer induces abortion of gametes with both alleles highly reducing the chance of its fixation in a population. In the study of *S5* sterility locus, the combination of non-functional Protector, and functional Killer and its partner (*ORF3*–, *ORF4*+, and *ORF5*+) was not found in the surveyed population (Yang et al., 2012). It may suggest that the combination that has Killer alone, without having functional Protector, cannot survive long in a population and also suggest that Killer can exist with functional Protector. However, after the emergence of Killer-Protector system, a single hybrid sterility locus with two tightly linked genes produces incompatibility when it meets the divergent allele in a different population. Then, the selfish nature of the system (i.e., TRD) caused by Killer-Protector system may facilitate its spread in a population.

## Discussion

### Evolution of Sex-Specificity in TRD in Rice

Approximately 50 hybrid sterility loci were identified in diverged species from the genus *Oryza*, and so far, cloning and characterization at a molecular level were already implemented for 10 loci out of those sterility loci or pairs (Ouyang and Zhang, 2013; Li et al., 2020). Among 49 hybrid sterility loci surveyed in this review, the number of loci for *m*TRD is the largest, in contrast to that of *fTRD* and *siTRD* (Table 1). In addition, the sterility based on gamete specificity disproportionately distributes between interspecific and intraspecific hybrids (Table 1). The distribution of *m*TRD loci is much wider in interspecific hybrids. On the other hand, all the *f* TRD loci are detected exclusively in intraspecific crosses. With regards to *siTRD*, it takes <15% of the total number of hybrid sterility loci, and it is very few in proportions compared to the other two sterility factors, *m*TRD and *f* TRD. Such a bias in frequency of sex-specificity in TRD system observed in *Oryza* might reflect different evolutionary pressures acting on the system, suggesting that TRD systems are not driven only by mutation and genetic drift. Therefore, uncovering the mechanisms underlying the observed pattern of TRD systems may be a key to understanding the evolution of reproductive isolation in *Oryza*. Although there is no direct evidence, several conjectures are possible to explain these patterns.

### The Abundance of *m*TRD

The simple difference between male and female gametes is the quantity of them produced by a single plant. In a single flower of rice, thousands of pollen grains are produced, while only one egg is produced. Such a difference in the quantity of gametes can cause differences in selection pressure acting on factors which induce the abortion of them. One can easily imagine that the reduction of pollen numbers causes less effect on fecundity than that of egg numbers. The PK, which is one factor causing *m*TRD, induces abortion of only pollen grains of a specific genotype. Therefore, *m*TRD caused by a PK can spread in a population with a small selective disadvantage. In contrast, female-specific TRD (*f* TRD) and sex-independent TRD (*si*TRD) induced by EK and GE, respectively, cause abortion of eggs. Thus, *f* TRD and *si*TRD offers a selective disadvantage on the individual. The abundance of male sterility in hybrids is also found in *Drosophila* (Presgraves and Meiklejohn, 2021). A much smaller selective disadvantage of male sterility genes might help them exist within diverse populations at a higher pace of frequency. However, we also note that other experimental factors might be able to explain the abundance of *m*TRD reported: pollen sterility is easier to detect/analyze than egg sterility because a large number of pollen grains are available for the assay.

### Uncommonly Observed Sex-Independent TRD

Among the three types of TRD systems, *si*TRD is less frequently observed (Table 1). In addition, most of them are observed in interspecific cross combinations. These observations suggest that hybrid sterility genes causing both male and female sterility occur less frequently and more time is necessary for fixation than ones causing single sex-specific sterility.

Although the *S1* locus for *si*TRD has been cloned, molecular mechanisms causing sex-independent sterility in pollen grains and eggs are unknown. Because pollen grains and eggs are developed in physically separated tissues (i.e., anthers and ovaries), it is difficult to imagine that abortion of gametes in one sex causes gametic abortion of another sex. The sex-independent abortion of gametes might be caused by disturbance of the biological/developmental process common in two sexes. Therefore, the rareness of *si*TRD might reflect less abundancy of the biological/developmental process common in two sexes than in one sex.

Another possible mechanism for the emergence of *si*TRD is a combination of factors for *m*TRD and *f* TRD. If the two genes, each of which causes *mTRD* and *fTRD*, respectively, are located in tightly linked regions on a chromosome, the region is expected to behave like a factor for *si*TRD. Because of the limited number of cloned loci for *si*TRD, it is still unknown how often such a “pseudo-*si*TRD” occurrs. In the case of the *S1* locus, which causes *si*TRD in inter-specific hybrids, Koide et al. (2008c) reported the change of sex-specificity of TRD depending on the length of introgressed chromosomal segments. The line with a long introgressed segment on chromosome 6 from *O. glaberrima* in the genetic background of *O. sativa* causes both pollen and embryo sac abortion when crossed with *O. sativa*. As a result, *si*TRD is observed in the next generation of hybrids. In contrast, when the line with a short introgressed segment from *O. glaberrima* was crossed with *O. sativa*, only pollen abortion and *m*TRD were observed. These results suggested the presence of two linked factors responsible for *m*TRD and *f* TRD in the region (We note that no other research groups have reported the change of sex-specificity of TRD induced by the *S1* locus.). Another locus, the *S6* for *si*TRD, has been suggested to be a compound locus of *f* TRD and pollen competition (Koide et al., 2012). In the *si*TRD induced by the *S6* locus, preferential abortion was observed in ovules, but not in pollen grains, suggesting that *m*TRD was caused by competition of pollens with different genotypes. The degree of TRD was altered only for male gametes when genetic background was changed. These results suggested the presence of two different genes for *m*TRD and *f* TRD in a closely linked region, though no direct evidence of these two factors were reported. As we described above, the gene for *m*TRD may be easier to evolve than that for *f* TRD, because of its small selective disadvantage on fecundity. Therefore, if the *si*TRD system originated *via* tight linkage between *m*TRD and *f* TRD, the rate of its emergence is dependent on how often *f* TRD evolved in the chromosomal region closely linked to the *m*TRD. It should also depend on the strength of recombination between these two factors in the chromosomal region.

Although the common genetic basis of *si*TRD (i.e., one factor or two factors) is still unknown, the evolution of *si*TRD is dependent on the balance between transmission advantage through pollen and disadvantage of female gamete abortion. Therefore, population size and outcrossing rate also affect the evolutionary process of *si*TRD. Uncovering the molecular basis and evolutionary trajectories of *si*TRD system will provide clearer insight into how selfish elements relate to the development of species' barriers as theorized by Frank (1991).

## Conclusions

In *Oryza* genus, reproductive isolation is excessively influenced by PK rather than EK and GE, which results in preferential occurrence of *m*TRD in contrast to the other two types, *f* TRD and *si*TRD. Compared to single sex-specificity, factors controlling *si*TRD are less frequently observed, mostly in interspecific hybridizations. Unveiling the underlying cause(s) behind this disproportionate pattern of TRD systems will shed light on the evolutionary process of reproductive barriers between rice relatives. Since our understanding on TRD systems remains very limited with confined experimental factors, further efforts are required to extend our investigation on many other selfish genes that exist and their distribution in *Oryza* genus.

## Author Contributions

Zin Mar Myint and YK contributed to conception, analyzed, and wrote the manuscript. All authors contributed to manuscript revision, read, and approved the submitted version.

## Funding

YK was funded by JSPS KAKENHI Grant Number 21H02160.

## Conflict of Interest

The authors declare that the research was conducted in the absence of any commercial or financial relationships that could be construed as a potential conflict of interest.

## Publisher's Note

All claims expressed in this article are solely those of the authors and do not necessarily represent those of their affiliated organizations, or those of the publisher, the editors and the reviewers. Any product that may be evaluated in this article, or claim that may be made by its manufacturer, is not guaranteed or endorsed by the publisher.
